# Exosome-Mediated Transfer of Cancer Cell Resistance to Antiestrogen Drugs

**DOI:** 10.3390/molecules23040829

**Published:** 2018-04-04

**Authors:** Svetlana E. Semina, Alexander M. Scherbakov, Anna A. Vnukova, Dmitry V. Bagrov, Evgeniy G. Evtushenko, Vera M. Safronova, Daria A. Golovina, Ludmila N. Lyubchenko, Margarita V. Gudkova, Mikhail A. Krasil’nikov

**Affiliations:** 1 N.N. Blokhin National Medical Research Center of Oncology, Kashirskoye shosse 24, Moscow 115478, Russia; s.e.semina@gmail.com (S.E.S.); vera.svm@gmail.com (V.M.S.); dar.golovina@gmail.com (D.A.G.); clingen@mail.ru (L.N.L.); gudkova@ronc.ru (M.V.G.); krasilnikovm1@ya.ru (M.A.K.); 2 Faculty of Preventive Medicine, I.M. Sechenov First Moscow State Medical University, Trubetskaya Street 8-2, Moscow 119991, Russia; biochem.fan@ya.ru; 3 Faculty of Biology, Lomonosov Moscow State University, 1/12, Leninskie gory, Moscow 119234, Russia; dbagrov@gmail.com; 4 Faculty of Chemistry, Lomonosov Moscow State University, 1/3, Leninskie gory, Moscow 119234, Russia; evtushenko@enzyme.chem.msu.ru

**Keywords:** SERM, breast cancer, exosomes, tamoxifen, resistance

## Abstract

Exosomes are small vesicles which are produced by the cells and released into the surrounding space. They can transfer biomolecules into recipient cells. The main goal of the work was to study the exosome involvement in the cell transfer of hormonal resistance. The experiments were performed on in vitro cultured estrogen-dependent MCF-7 breast cancer cells and MCF-7 sublines resistant to SERM tamoxifen and/or biguanide metformin, which exerts its anti-proliferative effect, at least in a part, via the suppression of estrogen machinery. The exosomes were purified by differential ultracentrifugation, cell response to tamoxifen was determined by MTT test, and the level and activity of signaling proteins were determined by Western blot and reporter analysis. We found that the treatment of the parent MCF-7 cells with exosomes from the resistant cells within 14 days lead to the partial resistance of the MCF-7 cells to antiestrogen drugs. The primary resistant cells and the cells with the exosome-induced resistance were characterized with these common features: decrease in ERα activity and parallel activation of Akt and AP-1, NF-κB, and SNAIL1 transcriptional factors. In general, we evaluate the established results as the evidence of the possible exosome involvement in the transferring of the hormone/metformin resistance in breast cancer cells.

## 1. Introduction

The efficiency of endocrine therapy of tumors, including breast cancer, is limited by the development of hormone-independent tumors which are resistant to antiestrogens initially or acquire resistance under prolonged therapy with antiestrogens (tamoxifen, raloxifene) [1,2,3]. The mechanism of hormonal resistance was investigated thoroughly. The main ways of the progression of hormonal resistance were found to include the loss or dysregulation of estrogen receptors, stimulation of growth-dependent pathways and activation of epithelial-mesenhymal transition, etc. [1,4,5,6,7,8,9].

The role of the intercellular interactions in the progression of hormonal resistance is less researched. Only a few studies demonstrate the effect of the horizontal transfer of the resistance between the cells, mainly of the development of multidrug resistance. Namely, the effect of the cell-to-cell transfer of functional p-glycoprotein occurs during co-cultivation of the parent and doxorubicin-resistant MCF-7 cells [10]. Furthermore, the cell ability to transfer the drug or tamoxifen resistance was shown to be mediated via exosomes secreted by the resistant cells [11,12] as well as via several exosomal microRNA or mRNA [11,13,14,15]. However, the role and significance of cell-cell interactions and/or exosomes in the mediating and formation of hormonal resistance of tumor cells is still not clear.

Exosomes are small sized vesicles that are generated in the cells and released into the extracellular space. They usually contain the various molecules including nucleic acids, proteins, lipids, etc. The growing interest in exosomes is based on their ability to shuttle from one cell to another and deliver biomolecules incorporating into the recipient cells [16,17,18,19]. It was found that the influence of exosomes on the cells may be implemented due to genomic or epigenomic changes. The first one can be caused by the integration of exosomal DNA into the host DNA. The second one—via the modulation of the content and/or activity of the signaling proteins, microRNA, etc. Undoubtedly, one of the most perspective achievements in the exosome study is the demonstration of their ability to provide the horizontal transfer of the genetic information between cells; this has been supported by the different studies with the various cell models [14,15,20]. 

The main goal of the present work was to study the mechanism of the interactions between hormone-sensitive and resistant cells, and exosome involvement in the progression of hormonal resistance. Earlier we had demonstrated for the first time that the effect of horizontal transferring of hormonal resistance of breast cancer cells was provoked by the co-cultivation of the hormone-sensitive cells and the resistant cells, and revealed the key proteins involved in the progression of the resistance [21]. Furthermore, we have found that tamoxifen resistance of breast cancer cells may be accompanied with the cross-resistance to cytostatic action of metformin, an anti-diabetic drug. Metformin exhibited the marked anti-tumor activity as well as the ability to inhibit the estrogen signaling [22].

Here, using two resistant MCF-7 sublines: the estrogen-independent subline MCF-7/T developed by the long-term treatment with antiestrogen tamoxifen, and metformin-resistant subline MCF-7/M—developed by the treatment with metformin, we have demonstrated the effect of cell cross-resistance to both drugs, showed the involvement of exosomes in the horizontal transferring of hormonal/metformin resistance in breast cancer cells, and revealed the resistant-associated signaling pathways affected by exosome treatment.

## 2. Results

### 2.1. Development and Characteristics of MCF-7 Resistant Derivatives

**Figure 1:**
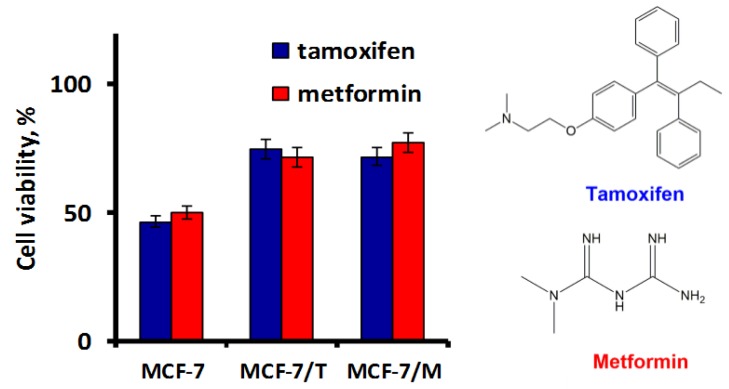
The characteristics of the resistant cell sublines. Cell sensitivity to tamoxifen and metformin. The cells were treated with 5 μM SERM tamoxifen or 10 mM biguanide metformin for 3 days and the amount of the viable cells was assessed by the MTT-test. Data represent mean value ± S.D. of three independent experiments. 100% was set as the viability of cells treated with vehicle control.

Previously we have shown that long-term metformin treatment of the parent MCF-7 cells resulted in the irreversible inhibition of estrogen signaling and cross-resistance to cytostatic action of metformin and antiestrogen tamoxifen [22]. To further explore the relations between acquired resistance to metformin and tamoxifen, we compared the sensitivity of two independent MCF-7 sublines: MCF-7/T subline developed by long-term tamoxifen treatment and MCF-7/M subline generated under metformin treatment, to these drugs. As shown, both sublines demonstrated the cross-resistance to tamoxifen and metformin cytostatic action (Figure 1).

Progression of hormonal resistance may be associated with the selection of the cells with the growth-related mutations, particularly, with the mutations in the genes of tyrosine kinase cascade and PI3K signaling [23]. Is the development of the resistant MCF-7 sublines, MCF-7/T and MCF-7/M, associated with the selection of the mutant cells? To study this possibility, the next generation sequencing (NGS) of the key genes: PIK3CA, EGFR, EGFR-AS1, ESR1 and ALK, was performed. Analysis of the parent MCF-7 cells revealed the coding mutation in PIK3CA only, in agreement with COSMIC [24]. The study of the resistant cells, MCF-7/T and MCF-7/M, revealed the presence of the parent PIK3CA mutation with non-changed frequency in both sublines; the coding mutations in other genes were not found (Table 1). Taken together, the results demonstrated that in vitro development of the tamoxifen/metformin resistant sublines was not connected with the selection of the cells with the preexisting or generated de novo mutations in the driver genes. 

### 2.2. Purification and Characterization of the Exosomes

**Figure 2:**
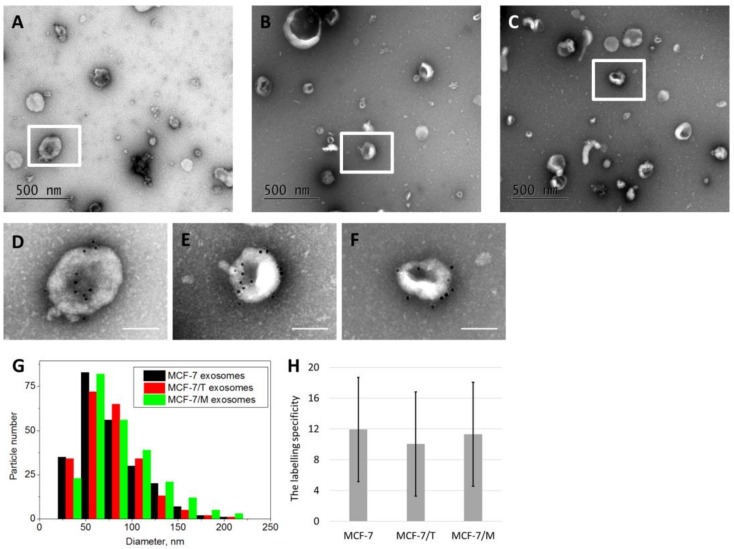
The transmission electron microscopy of the exosomes. Exosomes were prepared from the conditioned medium by the differential ultracentrifugation, labeled by the gold nanoparticles, and imaged as described in Methods. (**A**–**C**) Wide-field images of the exosomes from MCF-7, MCF-7/T and MCF-7/M cells correspondingly. (**D**–**F**) The magnified fragments, scale bar 100 nm. (**G**) Exosome size distributions obtained by processing of the TEM images. (**H**) The labelling specificity of the exosomes in the three samples obtained from MCF-7, MCF-7/T and MCF-7/M cells. The error bars correspond to S.D.

Exosomes were prepared from the MCF-7, MCF-7/T and MCF-7/M’ conditioned medium by the differential ultracentrifugation, and exosome imaging was carried by transmission electron microscope as described in Methods. The appropriate controls were included in all exosome functional studies as recommended by ISEV in Ref. [25] and by Takov K. et al. in Ref. [26]. The major part of the exosomes was either round, or had a cup-shape morphology. Figure 2A–F shows the images of exosomes obtained by TEM. The exosome size was in the typical range from 50 to 200 nm (Figure 2G). The exosomes obtained from MCF-7, MCF-7/T and MCF-7/M cells demonstrated similar ability to bind gold particles (Figure 2H).

**Figure 3:**
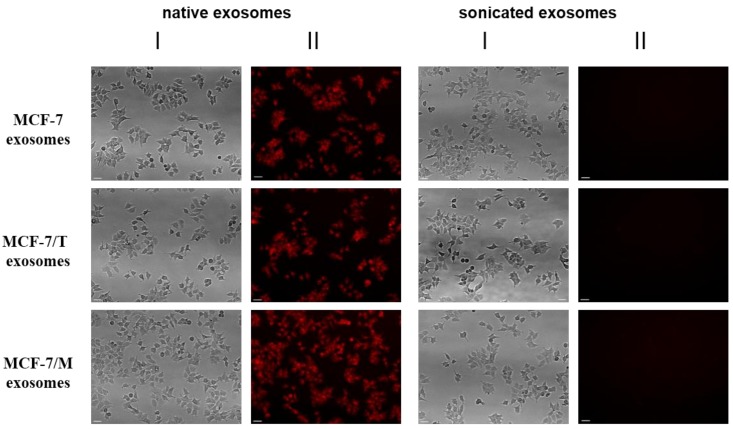

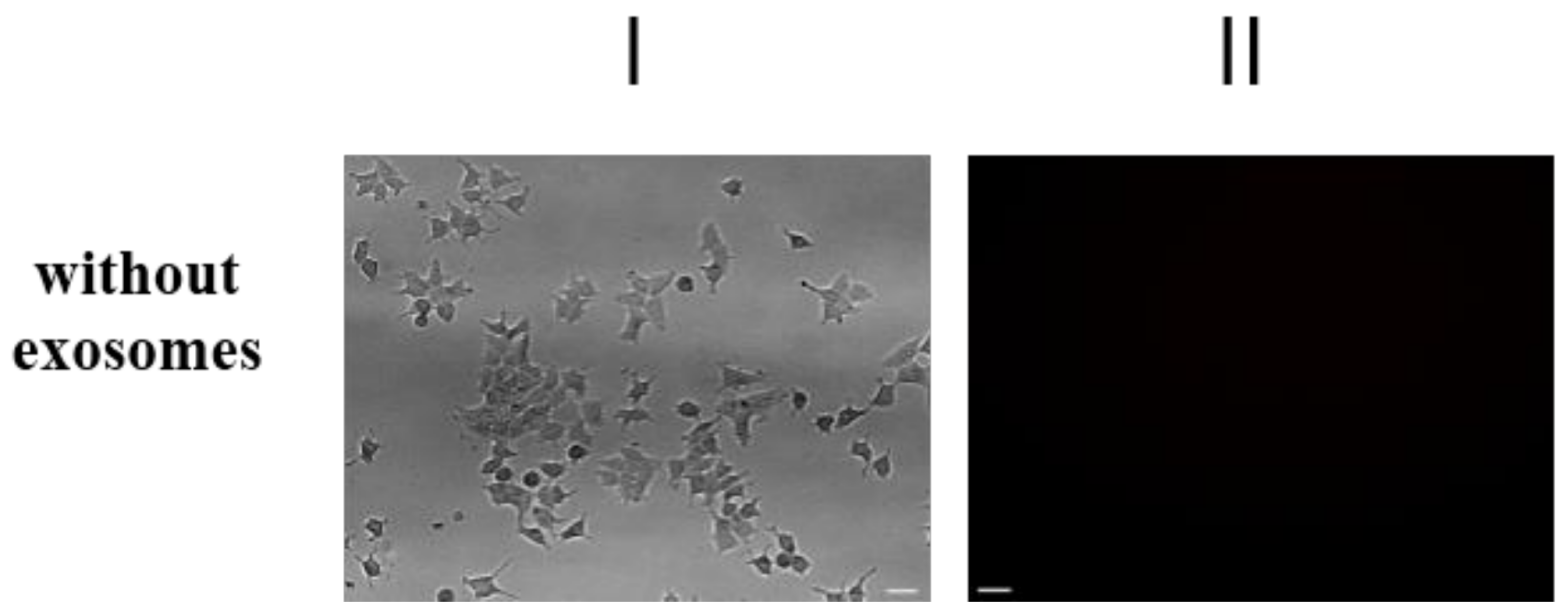
Transferring of fluorescent-labeled compounds by exosomes. Exosomes were stained by fluorescent drug (CellTracker™ Red CMPTX Dye) in according to the manufacturer’s procedure, then washed twice by the ultracentrifugation 100,000× *g* and incubated with MCF-7 cells. As a control labeled exosomes after sonication were used. The non-specific labeling of cell was checked by the fluorescent dye which was spun alone. The efficiency of dyeing exosome incorporation was checked with fluorescent microscope Nikon Eclipse Ti-E (Plan 10×/0.25; ORCA-ER camera by Hamamatsu Photonics; NIS-Elements AR 2.3 software by Nikon). Exposition for fluorescence was 4 s. Scale bar 50 µm. The images of light (I) and fluorescent (II) microscopy are presented.

The ability of extracellular vesicles to transport and incorporate to recipient cells was measured using fluorescent-labeled dye (CellTracker™ Red CMPTX Dye) as described in the Methods section. Exosomes were tagged and incubated with cells for 60 min. As a negative control, the sonicated samples of exosomes and fluorescent dye spun alone without exosomes were used. Figure 3 showed that the native (not sonicated) vesicles were able to accumulate the fluorescent drug and transfer it to recipient cells.

**Figure 4:**
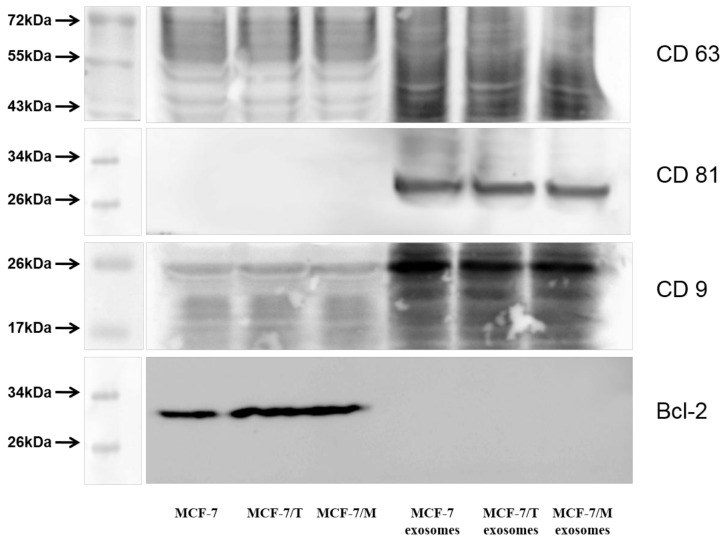
Immunoblotting of exosomal markers CD9, CD63, CD81 in the exosome samples from MCF-7, MCF-7/T and MCF-7/M cells versus cell lines MCF-7, MCF-7/T and MCF-7/M. As a non-exosomal marker was chosen Bcl-2 protein. The blot represents the results of one of the three similar experiments. The western blot analysis of exosome samples versus cell included non-reducing condition and a sample buffer did not contain β-mercaptoethanol.

The analysis of exosome preparations by western blotting revealed the key exosomal markers: CD9, CD63, CD81 in all samples. In order to demonstrate the purity of the preparation we used non-exosomes marker Bcl-2 in studied cell lines MCF-7, MCF-7/T and MCF-7/M (Figure 4) as recommended in [25]. 

The samples were normalized by protein content. Quantification of exosomes was also performed by nanoparticle tracking analysis (NTA). Exosomes were prepared from 3 independent passages of each subline. Exosome concentrations varied from 0.8 to 3.2 × 10^11^ vesicles/mL, mean particle size ranged from 129 to 179 nm in reasonable agreement with the results obtained by TEM. We attribute these variations of size and concentration to varying efficiency of exosomes pellet resuspension in PBS after the high-speed centrifugation. Nevertheless the particle concentration was proportional to protein concentration: С(particles/mL) = k × C(protein) with R^2^ = 0.95. CI_95_ for k was calculated to be (3.3 ± 0.2) × 10^9^ vesicles per µg of exosomal protein. This coefficient was further used for calculation of exosomes dosage.

### 2.3. Exosomes Influence on the Cell Response to Tamoxifen and Metformin

**Figure 5:**
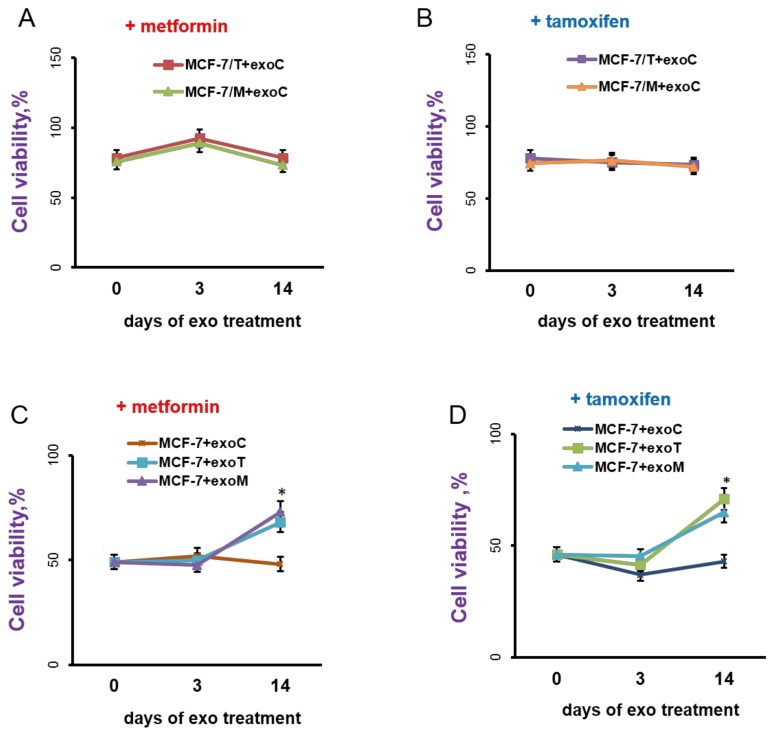
Exosomes influence on the cell response to metformin and tamoxifen. (**A**,**B**) The resistant MCF-7/T and MCF-7/M cells were cultured without exosomes or in the presence of the control exosomes from MCF-7 cells for 3 or 14 days, then the cells were treated with 5 μM tamoxifen or 10 mM metformin for 3 days and the amount of the viable cells was counted by the MTT-test. (**C**,**D**) The MCF-7 cells were cultured in the presence of the exosomes from MCF-7, MCF-7/T or MCF-7/M cells for 3 or 14 days, then the cell response to metformin and tamoxifen was determined as described above. Data represent mean value ± S.D. of three independent experiments. Сell viability (%) was expressed as a percentage relative to cells treated with vehicle control. * *p* < 0.05 versus MCF-7 + exoC.

The exosomes were prepared by differential centrifugation of the conditioned media after 3 days of cell growth as described in the Methods. Exosomes in PBS were added to 1.5 mL of cell suspension in a final concentration 1.7 μg/mL of exosomal protein or CI_95_ = (5.5 ± 0.3) × 10^9^ vesicles/mL once every three days at the time of splitting. Because the MCF-7/T and MCF-7/M cells demonstrate the cross resistance to tamoxifen and metformin (see Figure 1), the exosomes influence on the cell response to both drugs was analyzed. As shown, neither short-term (within 3 days) nor long-term (14 days) treatment of MCF-7/T and MCF-7/M cells with exosomes from the parent MCF-7 cells (exoC) changed the resistant properties of MCF-7/T and MCF-7/M cells: both sublines preserved the high resistance to tamoxifen and metformin (Figure 5A,B).

Whereas the treatment of the parent MCF-7 cells with exosomes from the resistant MCF-7/T or MCF-7/M cells (exoT and exoM, respectively) within 3 days did not affect the MCF-7 cells response to tamoxifen or metformin, the long-term exoT or exoM treatment (14 days) caused a marked decrease in the cell sensitivity to these drugs. Importantly, both exoM- and exoT-treated MCF-7 cells have acquired the cross-resistance to metformin and tamoxifen, when the exosomes from the parent MCF-7 cells (exoC) showed no effect on the cell response to the drugs (Figure 5C,D).

**Figure 6:**
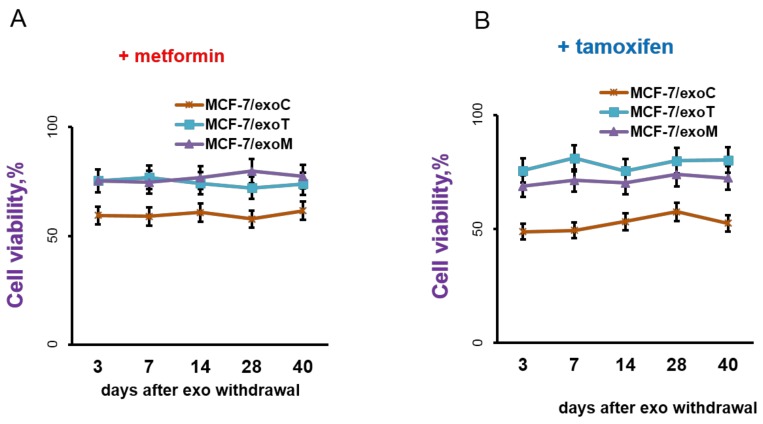
Exosome withdrawal and cell response to metformin and tamoxifen. MCF-7 cells after 14 days’ treatment by exosomes from MCF-7, MCF-7/T or MCF-7/M cells (named as MCF-7/exoC, MCF-7/exoT and MCF-7/exoM cells, respectively) were transferred to standard exosome-free medium and cell sensitivity to metformin (**A**) and tamoxifen (**B**) was regularly measured within 40 days of growth.

How long do the new-generated resistant properties persist in the exosome-treated cells? To answer this question, the MCF-7 cells, after 14 days of exosome treatment (named as MCF-7/exoC, MCF-7/exoT and MCF-7/exoM) were transferred to a standard exosome-free medium and cell sensitivity to drugs was regularly measured within 40 days of growth. The data showed no restoration of the cell sensitivity to the drugs in MCF-7/exoT and MCF-7/exoM cells demonstrating the irreversible character of such resistance (Figure 6). All subsequent experiments were performed using MCF-7/exoC, MCF-7/exoT and MCF-7/exoM cells after 40 days of exosome withdrawal.

### 2.4. Protein Signature of the Exosome-Generated Resistant Cells

**Figure 7:**
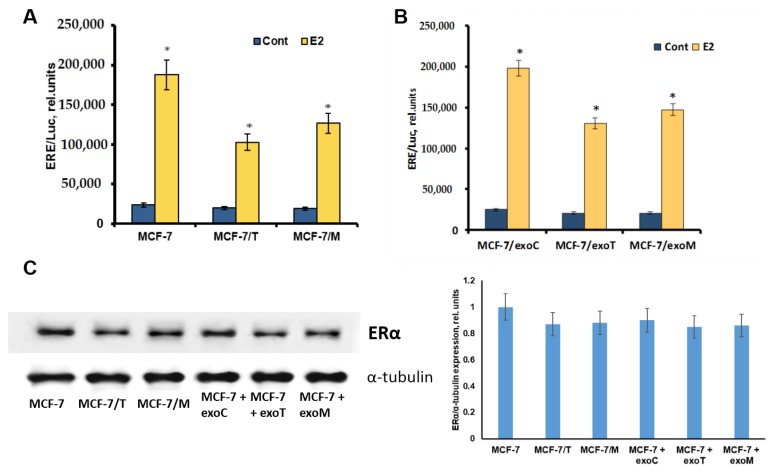
Expression and transcriptional activity of ERα. All experiments were performed on the donor MCF-7, MCF-7/T, MCF-7/M cells (**A**) and MCF-7/exoC, MCF-7/exoT, MCF-7/exoM cells treated with the respective exosomes for 14 days with following exosome withdrawal for 40 days (**B**). Transcriptional activity of ERα was determined by reporter assay. The cells were transfected with the ERE-LUC plasmid containing the luciferase reporter gene under the estrogen responsive element (ERE), and β-galactosidase plasmid. 24 h after transfection the luciferase and β-galactosidase activities were determined as described in the Methods section. The relative luciferase activity was calculated in arbitrary units as the ratio of the luciferase to the galactosidase activity. Data represent mean value ± S.D. of three independent experiments. * *p* < 0.05 versus control (-E2) (**C**) Western blot analysis of ERα in total cell extracts. Protein loading was controlled by membrane hybridization with α-tubulin Abs. The blot represents the results of one of the three similar experiments. Densitometry was performed using ImageJ (NIH) software with the protocol provided by The University of Queensland. Densitometry data represent mean value ± S.D. of three independent experiments.

**Figure 8:**
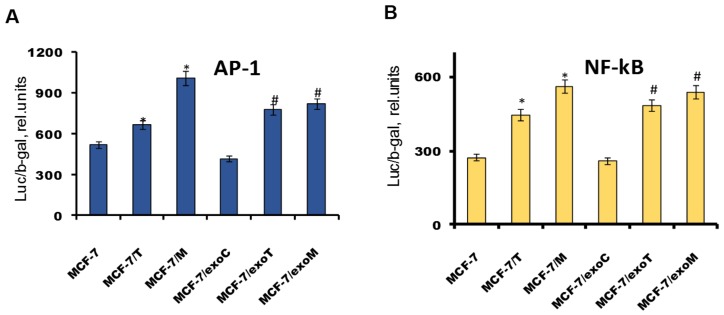
Transcriptional activity of AP-1 and NF-κB. The cell lines were similar to that in Figure 5. Transcriptional activity of AP-1 (**A**) and NF-κB (**B**) was determined by reporter assay. The cells were transfected with the AP-1 or NF-κB plasmid containing the luciferase reporter gene under the AP-1 or NF-κB-responsive elements, and β-galactosidase plasmid. 24 h after transfection the luciferase and β-galactosidase activities were determined as described above. Data represent mean value ± S.D. of three independent experiments. * *p* < 0.05 versus MCF-7, ^#^ *p* < 0.05 versus MCF-7/exoC.

To study the signaling pathways involved in the exosome-mediated resistance we have analyzed several growth-related transcription factors/proteins associated with hormonal resistance. As known, the progression of hormonal resistance is accompanied with the suppression of estrogen signaling and parallel activation of growth and epithelial-mesenchymal transition (EMT) pathways [9,27]. Comparative analysis of estrogen signaling revealed marked decrease in E2-mediated ERα transcriptional activity in the resistant cells: both in the donor MCF-7/T, MCF-7/M cells (Figure 7A), and in the exosome-treated MCF-7/exoT, MCF-7/exoM cells (Figure 7B), whereas the protein level of ERα was not changed significantly (Figure 7C). Furthermore, we compared the activity of AP-1 and NF-κB. Transcriptional factors AP-1 and NF-κB mediate the growth and anti-apoptotic effects, respectively, and, at the same time, exhibit the ability to interact with ERα [28,29]. Using the respective plasmid constructs containing the luciferase reporter gene under AP-1 or NF-κB-sensitive promoters, we found that the cells with exosome-mediated resistance (MCF-7/exoT, MCF-7/exoM) are characterized with marked activation of AP-1 and NF-κB—similar to that in the primary resistant cells (MCF-7/T, MCF-7/M) (Figure 8). 

**Figure 9:**
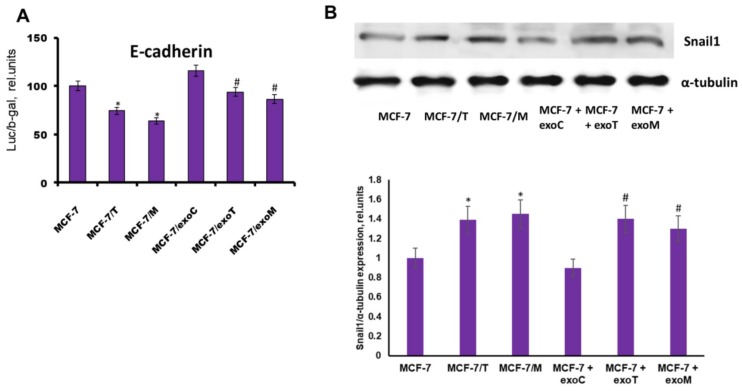
Expression and transcriptional activity of Snail1. The cell lines were similar to that in Figure 5. (**A**) Transcriptional activity of Snail1 was determined by reporter assay. The reporter assay was based on the Snail1 ability to inhibit the expression of the transfected luciferase reporter gene contained Snail-responsive elements from E-cadherin promoter (E-cad/Luc). The transfection efficiency was controlled by co-transfection of the cells with plasmid containing the β-galactosidase gene, luciferase activity was determined as described in the Methods section. Data represent mean value ± S.D. of three independent experiments. * *p* < 0.05 versus MCF-7, # *p* < 0.05 versus MCF-7/exoC. (**B**) Western blot analysis of Snail1 in total cell extracts. Protein loading was controlled by membrane hybridization with α-tubulin Abs. Densitometry was performed using ImageJ (NIH) software. Densitometry data represent mean value ± S.D. of three independent experiments. * *p* < 0.05 versus MCF-7, # *p* < 0.05 versus MCF-7 + exoC.

The parallel reporter analysis of E-cadherin promoter revealed its inhibition in all resistant cells (Figure 9A). Earlier we have shown that progression of tamoxifen/metformin resistance of MCF-7 cells was associated with the stimulation of EMT, and demonstrated the involvement of EMT-related SNAIL1 protein, one of the key down-regulators of E-cadherin, in the maintaining of the growth of the resistant cells [9]. Here the analysis of the SNAIL1 expression revealed the marked SNAIL1 activation in both the primary and exosome-induced resistant lines supporting the involvement of SNAIL1/E-cadherin signaling in the progression of resistance (Figure 9B).

**Figure 10:**
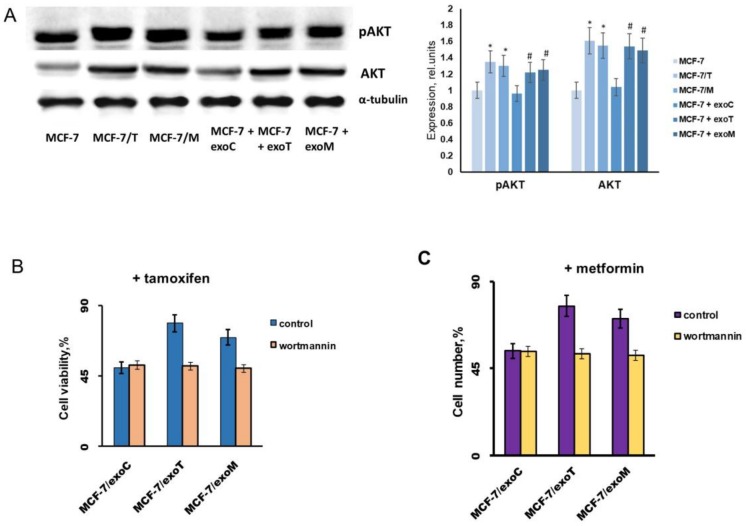
PI3K/Akt signaling and cell resistance. (**A**) Western blot analysis of pAkt and Akt in total cell extracts. The cell lines were similar to that in Figure 5. Protein loading was controlled by membrane hybridization with α-tubulin Abs. The blot represents the results of one of the three similar experiments. Densitometry was performed using ImageJ (NIH) software. * *p* < 0.05 versus MCF-7, # *p* < 0.05 versus MCF-7 + exoC. (**B**,**C**) Wortmannin influence on the exosome-mediated resistance. MCF-7 cells were treated with the control MCF-7 or “resistant” MCF-7/T and MCF-7/M exosomes within 14 days in the absence or presence 5 × 10^−6^ M wortmannin with subsequent determination of cell growth response to tamoxifen (**B**) or metformin (**C**).

As known, among the up-stream proteins involved in the regulation of growth/EMT-related pathways the central place belongs to the PI3K/Akt pathway [30]. The analysis of Akt showed a noticeable increase in both the total and phosphorylated (active) forms of this protein in all resistant lines (Figure 10A). To further explore the role of PI3K/Akt signaling in the progression of the resistance, MCF-7 cells were treated with the “resistant” (exoT and exoM) exosomes in the presence or absence of PI3K inhibitor wortmannin within 14 days. As shown, wortmannin completely prevented the progression of the exosome-induced resistance (Figure 10B,C).

In summary, the results obtained showed the similar rearrangement of the signaling pathways in the cells with primary resistance and in the cells with exosome-induced resistance, and demonstrated the important role of PI3K/Akt signaling in the exosome-transferring resistance.

## 3. Discussion and Conclusions

The main goal of this study was the analysis of the role of the intercellular interactions in the progression of hormonal resistance. The work was based on the hypothesis that the co-cultivation of the hormone-resistant and sensitive cells may lead to horizontal transfer of the hormonal resistance to the sensitive cells—as a result of the secretion of the specific factors, acting in the paracrine manner or via the direct cell-cell contacts. Previously, using estrogen-dependent MCF-7 cells and tamoxifen-resistant MCF-7/T subline we have shown that their co-cultivation led to irreversible resistance of the parent MCF-7 cells to tamoxifen [21], and the analogous effect was demonstrated for the metformin resistance. Our studies support several observations highlighting the effect of horizontal transferring of drug resistance between tumor cells. Namely, this effect was demonstrated for ABC-transporters, resistance-associated microRNA transferring [10,15,31,32], etc. Importantly, the cell ability to transfer the drug resistance was shown to be mediated via exosomes [11,12], demonstrating the key role of the latter in the resistance transfer. Concerning the tamoxifen resistance studies, the various types of microRNA were identified as proposal mediators of tamoxifen resistance [33] whereas only single observations demonstrate the involvement of exosomal microRNA, IncRNA or mRNA in the progression of hormonal resistance [13,14].

The present experiments were performed on in vitro cultured estrogen-dependent MCF-7 breast cancer cells and two resistant MCF-7 sublines: the estrogen-independent subline MCF-7/T developed by the long-term treatment of the cells with antiestrogen tamoxifen, and metformin-resistant subline MCF-7/M—developed by the long-term cell treatment with metformin (a biguanide antidiabetic drug, which exerts its anti-proliferative effect, at least in a part, via the suppression of estrogen machinery) [22]. As mentioned above, both developed sublines were able to transfer the resistant properties to the parent cells during long-term co-cultivation [21]. To further investigate the mechanism of the horizontal transferring of the resistance, the exosome involvement in the resistance development was studied.

As revealed, both sublines, MCF-7/T and MCF-7/M were characterized with the cross-resistance to tamoxifen and metformin. Importantly, the high-throughput sequencing of the driver growth-related genes (PIK3CA, EGFR, EGFR-AS1, ESR1 and ALK) showed no difference between the parent and resistant cells, supporting the possible involvement of the epigenetic mechanisms in the formation of the resistant phenotype. We found that the treatment of the parent MCF-7 cells with exosomes from the resistant MCF-7/T and MCF-7/M cells within 14 days lead to the partial resistance of the MCF-7 cells to both tamoxifen and metformin. At the same time, the exosomes from the parent MCF-7 cells did not affect the cell sensitivity to the drugs. What is interesting was that the exosome-induced resistance was not restored after exosome withdrawal—at least within 40 days of cell cultivation. One of the proposed explanations of this phenomenon may be connected with the acquired epigenetic modifications induced by exosomes—firstly, by exosomal microRNA. Actually, the evidences of the exosomal microRNA involvement in the regulation of various cell functions are increasing, and recent studies have demonstrated the important role of microRNA in the epigenetic regulation. Thus, miR-29b was found to be associated with suppression of DNMT3A/DNMT3 and a decrease in total DNA methylation [34,35], miR-143 was shown to be inversely correlated with DNMT3 expression [36]. Furthermore, total exosome preparations were found to affect the DNA methylation in the recipient cells [37]. On the other side, the correlation between progression of tamoxifen resistance and methylation status of growth-associated genes in breast cancer is well established and demonstrated on the various models including MCF-7 cells [38,39,40,41,42]. We propose that single exosomal microRNA may be involved in the epigenetic DNA modifications resulting in the partial cell resistance. However, further studies are required to explore the detailed mechanism of exosome action.

Generally, the progression of the hormonal resistance may be associated with the loss or decrease in the activity of estrogen receptors ERα accompanied with the constitutive activation of growth-related and anti-apoptotic pathways [6,7,8]. In our study, the analysis of the expression and activity of key signaling proteins revealed the marked changes in the proteins of the resistant cells. As revealed, both the primary resistant cells (MCF-7/T and MCF-7/M) and the cells with the exosome-induced resistance were characterized with common features: decrease in ERα activity and parallel activation of growth-dependent (AP-1), anti-apoptotic (NF-κB) and EMT-associated (SNAIL1) transcriptional factors. Totally, the results substantiate the existence of the common pathway targeted by the exosomes and activated in all resistant cells.

The PI3K/Akt pathway belongs to the central cellular pathways respondent for the growth/apoptosis regulation, including the maintenance of estrogen-independent growth [43]. We found marked increase in the expression and activity of Akt in all of the resistant cells compared with the parent MCF-7 cells. Furthermore, the cell treatment with PI3K inhibitor wortmannin prevented the exosome-induced resistance giving the additional evidence for a central role of this pathway in the mediating of exosomal resistance.

Another important point in the presented study was the mechanism of the overlapped resistance of breast cancer cells to tamoxifen and metformin. We showed that the exosome-mediated horizontal transferring of the resistance, from cell to cell, may occur: both for the classical variant of hormonal resistance caused by the long-term tamoxifen treatment, and for another variant of resistance caused by metformin treatment. As mentioned above, the cytostatic action of biguanide metformin on the breast cancer cells is accompanied with the irreversible inhibition of an estrogen receptor. We propose that common features of the tamoxifen-resistant (MCF-7/T) and metformin-resistant (MCF-7/M) cells—ERα suppression; activation of growth/EMT-related transcriptional factors; activation of PI3K/Akt signaling—may be caused by the metformin ability to suppress ERα resulting in the activation of the common (like in the tamoxifen case) pathways mediated by the exosomes. 

Further studies are required to analyze the cargo of the “resistant” exosomes—including the proteome and microRNA profile, and identify the key factors respondent for exosome-mediated transferring of the resistant phenotype.

## 4. Materials and Methods

### 4.1. Cell Cultures and Development of Drug Resistant Derivatives

The human breast cancer cell line MCF-7 (ATCC HTB-22™) was purchased from ATCC (Manassas, VA, USA). The cells were authenticated by morphology and STR profiling provided by Gordiz, (Moscow, Russia). Tamoxifen for cell cultures was purchased from Cayman Chemical (Ann Arbor, MI, USA), wortmannin and metformin were purchased from Merck KGaA (Darmstadt, Germany).

The tamoxifen-resistant MCF-7/T and metformin-resistant MCF-7/M sublines were established from the parent MCF-7 cells under prolonged tamoxifen or metformin treatment, respectively. Briefly, MCF-7 cells were cultured in DMEM medium containing 10^−6^ M tamoxifen or 2 × 10^−3^ M metformin for 6 months, then the cells were transferred to tamoxifen/metformin-free medium and subsequent growth of these cells was maintained in the absence of the drugs. The MCF-7 cell line and resistant MCF-7/T and MCF-7/M sublines were cultured in standard low glucose DMEM medium (Biolot, Saint Petersburg, Russia) supplemented with 7% fetal bovine serum (FBS) (HyClone, Logan, UT, USA) at 37 °C and 5% CO_2_. To determine the cell response to tamoxifen or metformin the cells were treated with 5 μM tamoxifen or 5 mM metformin for 3 days in standard DMEM medium with 7% FBS, and the amount of the viable cells was counted. 

The cell growth was evaluated by the modified MTT (3-(4,5-dimethylthiazol-2-yl)-2,5-diphenyltetrazolium bromide) (Applichem, Darmstadt, Germany) test [44] as described in [45]. Dimethylsulfoxide (DMSO) for MTT test was obtained from Applichem). Ultrapure water for experiments was prepared by Milli-Q water purification system (Millipore, Burlington, MA, USA). 

### 4.2. Next Generation Sequencing

#### 4.2.1. DNA Preparation

Samples for NGS were prepared using a DNA extraction kit produced by Qiagen(Hombrechtikon, Switzerland). MCF-7, MCF-7/M and MCF-7/T cells were grown for 72 h, washed twice in PBS and collected. DNA was isolated by a MagNa Pure (Roche Molecular Systems, Pleasanton, CA, USA) gadget following the manufacturer’s protocol. Quality control of isolated DNA was done by QIAxpert spectrophotometer (Qiagen) and RT-PCR. DNA concentration of each sample was higher than 100 ng/μL. The required amounts of diluent were added into the samples to obtain DNA concentration 2 ng/μL.

#### 4.2.2. Library Preparation for NGS. Clonal Amplification

The next stage was targeted enrichment when GeneReader Actionable Insights Tumor Panel was used and Enrichment by PCR was performed. Then preparation of libraries was carried out including ligation of adapters and amplification of libraries. After that clonal amplification (template preparation for sequencing) was done: amplification in emulsion and right after that monoclonal libraries on particles. After DNA library construction, DNA was clonally amplified using the GeneRead QIAcube (Qiagen) instrument, immobilized via direct bead-slide interaction, and exposed to a DNA sequencing primer to produce a high-density array on a GeneReader Flow Cell (Qiagen).

#### 4.2.3. Sequencing

DNA libraries were normalized by concentration followed by pooling. Sequencing stage divided into several steps: attaching particles to a cell and labeling nucleotides, the “splittable” terminators were used. The steps called attachment, image, cleaning. The array was scanned by a high resolution electronic camera and the fluorescent output of each of the 4 dye colors at each array position is measured and recorded. The color indicates which base (A, C, G or T) was incorporated onto the DNA fragment from the previous step. Finally, the array was exposed to cleavage chemistry to break off the fluorescent dye and end cap that will then allow additional bases to be added.

#### 4.2.4. NGS Mapping 

QCI Analyze and QCI Interpret software was used for data analysis. The software automatically performed a filtering of the fragments obtained on quality, adapter trimming and mapping of the fragments obtained according to reference genome hg19. Gene variants with statistical significance (*p* = 1.0 × 10^−4^) were selected by manual software.

For reliable evaluation of gene mutations 1.5% of tumor cells in the sample are enough for the analysis. The quality of the reads obtained after sequencing was higher than Q25 for more than 70% of studied DNA samples. The numbers of the reads for DNA samples analyzed after filtering were 1,490,465 for MCF-7, 1,195,387 for MCF-7/M and 1,376,487 for MCF-7/T. These data indicate the correctness of the normalization.

### 4.3. Transient Transfection and Measurement of Reporter Gene Activity 

To determine the transcriptional activity of Snail1, ERα, AP-1 and NF-κB the cells were transfected with the plasmids containing luciferase reporter gene controlled by Snail-binding element of E-cadherin promoter, canonical estrogen-responsive elements (ERE), AP-1 and NF-κB-responsive elements, respectively. The plasmids used in this work were kindly provided by Dr. Antonio García de Herreros, Dr. Victor Adler, Dr. George Reid, and Dr. Alexander Gasparian [46,47,48]. The transfection was carried out for 4 h at 37 °C using Metafectene PRO (Biontex, München, Germany). To this end, Metafectene PRO (0.8 µL) was complexed with 0.4 µg of DNA to transfect one well (24 well plates, Corning, NY, USA). To control the efficiency and potential toxicity of the transfection, the cells were transfected with the β-galactosidase plasmid. All subsequent experiments were performed during 24 h after transfection. The luciferase activity was measured according to a standard protocol (Promega, Madison, WI, USA) using an Infinite M200 Pro instrument (Tecan, Männedorf, Switzerland), and calculated in arbitrary units as the ratio of the luciferase/galactosidase activity as described in [49].

### 4.4. Western Blot Analysis of Cell Lysates

The cells were washed twice, and incubated for 10 min on ice in the total lysis buffer containing 50 mM Tris-HCl, pH 7.4, 1% SDS, 1% Igepal CA-630, 0.25% sodium deoxycholate, 150 mM NaCl, 1 mM EDTA, 1 mM PMSF; 1 µg/mL each aprotinin, leupeptin, pepstatin; 1 mM Na-orthovanadate and 1 mM NaF. Samples were sonicated 4 times for 5 s each at 30% output, centrifuged for 5 min at 15,000× *g*, and supernatants were then used as total cell extracts. Total protein content was determined by the Bradford method.

Cell lysates (40 µg protein) were separated in 10% SDS-PAGE under reducing conditions, transferred to a nitrocellulose membrane (SantaCruz, Dallas, TX, USA) and processed according to the standard protocol. To prevent nonspecific absorption, the membranes were treated with 5% nonfat milk (AppliChem) solution in TBS buffer (20 mM Tris, 500 mM NaCl, pH 7.5) with 0.1% Tween-20 and then incubated with primary antibodies overnight at +4 °C.

Primary antibodies to SNAIL1, (phospho)Akt, (Cell Signaling Technology, Kenilworth, NJ, USA) and ERα (Merck KGaA, Darmstadt, Germany)were used; the antibodies against α-tubulin (Cell Signaling Technology, Danvers, MA, USA) were used to standardize loading. Appropriate IgG’s (Jackson ImmunoResearch, West Grove, PA, USA) conjugated to horseradish peroxidase were used as secondary antibodies. Signals were detected by ECL reagent prepared as described in Mruk’s protocol [50] and ImageQuant LAS4000 system for chemiluminescence (GE HealthCare, Little Chalfont, UK). ImageJ software [51] was used for densitometry. 

### 4.5. Exosomes Purification and Analysis

#### 4.5.1. Exosomes Isolation by Ultracentrifugation

9 × 10^6^ MCF-7 or MCF-7/T cells were seeded and grown during 72 h on a triple-layers flasks T500 (Thermo Fisher Scientific, Waltham, MA, USA) in 100 mL DMEM medium with 7% FBS. To avoid contamination of samples by bovine serum exosomes the FBS was spun for 12 h at 100,000× *g* in advance. The control centrifugation of the prepared DMEM/7% FBS medium showed pellet protein concentration less than 5% comparing with exosome samples. Then exosomes from supernatant were isolated by differential ultracentrifugation using standard protocol [52]. Briefly, in order to eliminate dead cells and debris the supernatant was spun by successive centrifugation at increasing speed: 30 min 300× *g*, 30 min 2000× *g* and 30 min 8000× *g*. The final ultracentrifugation was 90 min 100,000× *g* (Koki CP 80NX, Hitachi, Chiyoda-ku, Tokyo, Japan). The pellets were washed in 200 μL of PBS. All procedures were sterile. 

##### Western Blot Analysis of Exosomes

The western blot analysis of exosomes or cell samples (10 µg protein) was proceeded as described above. For exosome detection, primary antibodies to CD9 (Millipore), CD63, CD81a (BioLegend, San Diego, CA, USA) were used. Total protein content was determined by the Bradford method and used to standardize loading. Importantly, the analysis of exosome samples versus cell included non-reducing condition and a sample buffer did not contain β-mercaptoethanol.

#### 4.5.2. Transportation of Fluorescent-Labeled Compounds and Peptides

In order to check the exosomes ability to incorporate to recipient cells the vesicles were labeled by fluorescent dye CellTracker™ Red CMPTX Dye (Thermo Fisher Scientific). After ultra-centrifugation exosomes were dissolved in PBS solution and stained by fluorescent dyes, according to the manufacturing protocols. Then thoroughly stained exosomes were washed in PBS twice by the ultracentrifugation 100,000× *g*. As a negative control the labeled exosomes were sonicated. In order to detect the non-specific labeling of cells we used the fluorescent dye which was spun alone. The precipitates were dissolved in PBS and incubated with MCF-7 cells. The efficiency of dyeing exosomes incorporation was checked with an Eclipse Ti-E fluorescence microscope (Nikon, Minato-ku, Tokyo, Japan) (Plan 10×/0.25; ORCA-ER camera by Hamamatsu Photonics, Minato-ku, Tokyo, Japan; NIS-Elements AR 2.3 software by Nikon). Exposure for fluorescence was 4 s. 

#### 4.5.3. Extracellular Vesicles Size and Concentration Measurements

Measurements of size and concentration for purified exosomes were made with nanoparticle tracking analysis (NTA) in accordance with ASTM E2834—12 [53]. Measurements of size and concentration for purified exosomes were made with nanoparticle tracking analysis (NTA) in accordance with ASTM E2834—12 [53] using a Nanosight LM10 HS-BF instrument (Nanosight Ltd., Salisbury, UK). Laser unit with 405 nm, 65 mW laser and high sensitivity EMCCD camera (Andor Luca, Belfast, UK) were used. Briefly, the sample was diluted with particle-free PBS to reach the concentration of around 1.5 × 10^8^ particles/mL (800–5000 times). 12 to 18 videos 60 s long were recorded for each sample to reach the total 4200–7100 individual tracks. Videos were processed with NTA software 2.3 build 33. Results from all measurement of the same sample were joined to calculate the mean hydrodynamic diameter and total particle concentration, corrected for dilution factor.

#### 4.5.4. Transmission Electron Microscopy (TEM)

TEM with immunogold labelling was used to visualize the exosomes samples. The carbon-coated TEM grids (Ted Pella, Redding, CA, USA) were treated with a glow discharge device Emitech X100K (Quorum Technologies Ltd., Laughton, Great Britain) to hydrophilize the carbon surface and increase the adsorption. The exosomes were deposited onto the grids for 5 min and rinsed with PBS. The surface was blocked with 50 mg/mL BSA to minimize the non-specific binding. The samples were incubated in 10 μg/mL solution of anti-CD9 (Millipore) antibodies for 30 min. Then the samples were rinsed with PBS and labelled by the 10 nm gold particles coated with protein A (Electron Microscopy Sciences, Hatfield, PA, USA). After 20 min labelling and the final rinse with PBS the samples were contrasted with 1% uranyl acetate and dried. Imaging was carried out using a JEM-1011 transmission electron microscope at 80 kV. At least 30 images were obtained for the exosomes of each type. Fiji software [54] was used to measure the exosome sizes. Immunogold labelling implies introduction of the gold nanoparticles (labels) into the samples. The TEM images of the labelled exosomes were analyzed to address the labelling specificity. The surface densities of the exosome-bound labels N_spec_ and the background non-specifically bound labels N_Bckg_ were used to estimate the labelling specificity. The ratio of the mean values <N_spec_>/<N_Bckg_> was at least 10 for all the studied samples, indicating the high specificity of the labelling procedure.

#### 4.5.5. Cells Treatment with Exosome Preparations

Cells were seeded on a 24-well plate (Corning). Exosomes were added before the cells attached to the plate. Concentration of exosomes was determined by NTA, protein concentration was evaluated by Bradford reagent (Merck KGaA, Darmstadt, Germany). Exosomes in PBS were added to 1.5 mL of cell suspension in a final concentration 1.7 μg/mL of exosomal protein or CI_95_ = (5.5 ± 0.3) × 10^9^ vesicles/mL once every three days at the time of splitting.

### 4.6. Statistical Analysis

Each experiment was repeated three times with three technical replicates. Statistical analysis was performed using Microsoft Excel (Microsoft, Redmond, WA, USA). Results were expressed as mean ± S.D. (standard deviation value) if not stated explicitly. *p* value of < 0.05 was considered to be statistically significant.

## Figures and Tables

**Table 1:** Coding mutations in MCF-7, MCF-7/M and MCF-7/T cells.

	Cell Line	MCF-7	MCF-7/M	MCF-7/T
Gene				
PIK3CA		c.1633G > A Allele fraction: 66% (of 17,311 reads)	c.1633G > A Allele fraction: 67% (of 12,976 reads)	c.1633G > A Allele fraction: 65% (of 11,224 reads)
ALK		NO	NO	NO
EGFR		NO	NO	NO
EGFR-AS1		NO	NO	NO
ESR1		NO	NO	NO

## Abbreviations

MTT	3-(4,5-dimethylthiazol-2-yl)-2,5-diphenyltetrazolium bromide
DMSO	Dimethylsulfoxide
STR	Short Tandem Repeats
DMEM	Dulbecco’s Modified Eagle Medium
ERE	Estrogen-responsive elements
FBS	Fetal Bovine Serum
SDS	Sodium Dodecyl Sulfate
TBS	Tris-Buffered Saline
E2	17β-Estradiol
